# Testing for Consistency in Co-occurrence Patterns Among Bacterial Taxa Across the Microbiomes of Four Different Trematode Parasites

**DOI:** 10.1007/s00248-025-02545-w

**Published:** 2025-05-17

**Authors:** Ryota Hasegawa, Robert Poulin, Priscila M. Salloum

**Affiliations:** Department of Zoology, University of Otago, P.O. Box 56, Dunedin, 9054 New Zealand

**Keywords:** Community assembly, Microbiome, Priority effects, Parasite, Trematode

## Abstract

Elucidating the specific processes and drivers of community assembly in the host microbiome is essential to fully understand host biology. Toward this goal, an important first step is to describe co-occurrence patterns among different microbial taxa, which can be driven by numerous factors, such as host identity. While host identity can be an important influential factor on co-occurrence patterns, a limited number of studies have explored the relative importance of host identity after controlling for other environmental factors. Here, we examined microbial co-occurrence patterns in four phylogenetically distinct trematode species living within the same snail species, collected concomitantly from the same habitat. Our previous study determined that all these trematodes shared some bacterial taxa, and the relative abundance of microbial taxa differed among trematodes, possibly due to differences in their eco-physiological traits. Here, we specifically predict that pairwise microbial co-occurrence patterns also vary among trematode host species. Our results showed that co-occurrence patterns among eight microbial families varied greatly among the four trematode hosts, with some microbial families co-occurring in some trematode species, whereas no such patterns were observed in other trematodes. Our study suggests that the habitat identity (trematode species) and its associated biotic characteristics, such as physiological and ecological traits, can determine co-occurrence patterns among microbial taxa, with substantial effects on local community composition.

## Introduction

It is now well recognized that individual organisms have a microbiome, i.e., complex communities of bacteria and other microorganisms, including all their genomes and gene products [1]. Microbiomes are essential factors affecting the phenotypes of living organisms, such as immunity [2], behavior [3], and life-history traits [4], ultimately driving a variety of ecological and evolutionary processes [5]. For these reasons, there has been growing interest in identifying the specific processes and drivers underpinning microbial community assembly.

Understanding the co-occurrence patterns among particular microbial taxa, which can represent the smallest subsets of the microbial community, is a crucial first step in elucidating the processes and drivers of microbial community assembly [6]. Indeed, many studies have explored the co-occurrence patterns of microbial taxa, in which either positive or negative correlations were often described (e.g., [7, 8]). When some microbial taxa use similar resources and/or prefer specific physical and physiological environments within the hosts, these taxa often co-occur within host individuals, showing positive correlations between their abundances across different individual hosts [8, 9]. Co-occurrences are also observed when a microbial taxon facilitates the establishment of another taxon by changing the environment within the hosts (e.g., host physiology) or producing another resource, which is beneficial for the other taxon [10]. In contrast, when microbial taxa prefer different environments, they are less likely to co-occur within the hosts, resulting in negative correlations in their abundance across host individuals [8]. Competition often occurs in microbial communities, especially when shared resources (e.g., space, food) are limited and/or one microbial taxa changes the environment, which inhibits establishments by others [8, 11, 12]. Therefore, overall, co-occurrence patterns among different microbial taxa can be driven by numerous processes and factors, making it difficult to identify the relative importance of these factors in shaping microbial co-occurrence patterns.

Among the many factors potentially affecting microbial co-occurrence patterns, host identity (i.e., host individuals or species) has been considered one of the most influential factors because each host species or individual exhibits different physical, physiological, and ecological characteristics, all of which could affect the filtering of certain microbial taxa [13–15]. Indeed, some studies found that microbial community structure and co-occurrence patterns differ among different hosts [13–17]. However, studies examining co-occurrence patterns among the same microbial taxa but within different host species are still limited (e.g., [13–15]). Furthermore, most earlier studies did not fully control the potential effect of external environmental conditions, making it impossible to identify whether host identity or other factors are important in microbial community composition and co-occurrence patterns (e.g., [13–17]).

Here, we examined co-occurrence patterns among different bacterial families shared across the same life-stage of four different trematode species infecting the same snail species in the same locality. This study system allows for a robust control of external environmental conditions: each individual trematode hosting a microbiota occurs in a different individual of the same snail species collected from the exact same locality on the same day [18]. This provides us with an ideal opportunity to test whether and to what extent microbial co-occurrence patterns vary among distinct hosts (i.e., trematode species). We predicted that co-occurrence patterns among microbial taxa would vary among trematode species because the trematode species are phylogenetically, ecologically, and physiologically different from each other, although they infect the same snails at the same time and place. In contrast, the opposite pattern (i.e., consistent direction and strength of co-occurrence patterns among bacterial taxa in all four trematodes) would suggest that co-occurrence patterns are only weakly affected by their immediate environment (i.e., the identity of the trematode hosting them). The four focal trematode species (*Galactosomum otepotiense*, *Philophthalmus attenuatus*, *Acanthoparyphium* sp., and *Maritrema novaezealandense*) utilize the same mud snails *Zeacumantus subacarinatus*, as their first intermediate hosts, and then mature as adults in shore birds. Our previous study found that the overall composition of their microbiota differs significantly [18]. Different ecological (i.e., feeding modes) and physiological (i.e., metabolism and immunity) traits of each trematode species might affect the composition of their microbiota [18]. Such differences among trematodes might also shape co-occurrence among the bacterial taxa they harbor. Despite overall differences among their microbiotas, several bacterial taxa occur across all four trematodes. Our findings provide a test of co-occurrence patterns, which can be important for inferring community assembly in bacterial assemblages, distinguishing between strong effects of immediate environmental conditions (variable pairwise associations among different trematode species) versus a strong and context-independent pattern of associations (consistent pairwise associations among different trematode species).

## Methods

### Study Species

We used the larvae (rediae/sporocycts) of the following four trematode species: *Galactosomum otepotiense* (Heterophyidae), *Philophthalmus attenuatus* (Philophthalmidae), *Acanthoparyphium* sp. (Himasthlidae), and *Maritrema novaezealandense* (Microphallidae) [19]. All four species utilize the mud snail *Zeacumantus subacarinatus* as their first intermediate hosts [19]. Within the snail hosts, trematodes are in an asexually reproductive larval stage (see below), which produce large numbers of free-swimming infective stages, or cercariae. These emerge from the snail to seek and infect various types of second intermediate hosts; *G. otepotiense* use fish [19], *P. attenuatus* use mollusks and crustaceans [19], *Acanthoparyphium* sp. use mollusks [19], and *M. novaezealandense* uses several crustaceans, such as amphipods [19]. After their second intermediate host is ingested, all four trematodes mature in sea birds, such as gulls, and sexually reproduce within these definitive hosts [19]. Eggs are released with feces and bodily fluids, and these eggs are ingested by mud snails. A single ingested egg develops into a larva, which then proceeds to multiply asexually within the snail, and the cycle continues. This asexually reproducing mass of larvae of the same species within one snail represents one individual sample in the following analyses.

### Sampling and Microbial Extractions

All datasets used in this study were generated in a previous study [18]. In brief, mud snails *Z. subcarinatus* were sampled at low tide in Lower Portobello Bay, Dunedin, New Zealand (45° 4,904,800 S, 170° 4,001,200 E) on the same day in March 2022. Sampled snails were brought back to the laboratory and kept alive with seawater collected at the sampling site. They were individually placed in wells on sterile tissue culture plates with seawater from the collection site and incubated at 25 °C for 24 h to identify infected individuals. Then, plates were screened for released cercariae under a dissecting microscope. When released cercariae were found, they were identified based on their morphology, and snails infected with each trematode species were sorted into different containers until they were dissected.

Sterile procedures were used for all dissections and laboratory work [18]. Five asexually reproducing larvae were extracted from each snail, and to avoid contamination from the snail host, larvae were washed thoroughly with sterile PBS by pipetting up and down 20 times in each of three wells of a culture plate [18]. The five larvae were pooled for DNA extraction, purification, and microbiome characterization, which was done by sequencing a fragment of the 16S SSU rRNA prokaryotic gene with the primers 515 F-806R [20, 21]. Resulting de-multiplexed sequences were checked for quality with FastQC v. 0.11.9 [22]. Plugins in Qiime2 v. 2021.4 [23] were used to process the sequences bioinformatically, denoising with the dada2 plugin [24], and conservatively filtering contamination (removing all sequences found in negative controls, mitochondria, chloroplasts, eukaryotes, and sequences failing phylum assignment). Data quality was evaluated by analyses of microbial community standards (see [18] for a more detailed description and specific results). Taxonomy was assigned to DNA sequences using the Silva SSURef_NR99 prokaryotic database version 138.1 (see [18] for more details). Relative bacterial taxon abundance (based on counts of DNA sequences) was calculated with the R package microeco 0.11.0 [25], and shared families among the four trematode species were identified with a Venn diagram [18]. Because of constraints in the level of taxonomic resolution achieved with only a small fragment of DNA (240 base pairs in the v4 region of the 16S rRNA prokaryotic gene), taxonomic assignment below family level was not always possible in this dataset [18]. In addition, many low-abundance genera and species were only found in one or a few individual trematodes [18]. Therefore, the microbial family level is the most appropriate taxonomic level for our study objective (i.e., testing whether co-occurrence patterns among different microbial taxa are consistent across the four trematode species). Additionally, complex bacterial functions have been shown to be evolutionary conserved [26, 27]. Thus, microbial species and genera within the same family are likely to have similar functions, at least for more complex traits [26–28]. See Salloum et al. [18] for detailed information regarding dissection, DNA extractions, and bioinformatics.

### Data Treatment

In the original study, some snails were co-infected with more than one trematode species; because coinfection potentially affects trematode microbiomes through horizontal transmission [18, 29], we excluded co-infected snails from the analysis below to minimize factors that could affect microbial interactions. This resulted in 22 *Maritrema novaezealandense*, 9 *Acanthoparyphium* sp., 10 *Galactosomum otepotiense*, and 12 *Philophthalmus attenuatus* individuals; each of these trematode individuals represents a replicate bacterial community. Bacterial families shared among all four trematode species were identified (matching Fig. 2D of [18], with 28 shared families). However, most of these families had variable relative abundances among conspecific trematode individuals, with many families showing values essentially at zero in many individuals, while being abundant in very few others (see [18]). Since our main objective in this study is to test whether pairwise associations between microbial families were consistent within the four trematode species, only bacterial families present in ~ 20% or more of the individuals of each trematode species were included (at least four individuals of *Maritrema*, and at least two individuals of the other three species). Consequently, the filtered dataset contains eight bacterial families (Burkholderiaceae, Comamonadaceae, Corynebacteriaceae, Hymenobacteraceae, Microbacteriaceae, Oxalobacteraceae, Rhodobacteraceae, Sphingomonadaceae) and 53 trematodes, each representing a distinct bacterial community.

### Statistical Analysis

To test whether co-occurrence patterns among microbial families are observed in each environment and context (i.e., across trematode species), and whether these co-occurrence patterns are consistent among host identities, we conducted a two-step hierarchical analysis. First, we tested for correlations of relative abundance between all pairs of microbial families within each trematode species to assess whether co-occurrence patterns among microbial families are consistent across environments and contexts (i.e., across trematode species). This involved examining correlations among 8(8 − 1)/2 = 28 combinations of different microbial families for each trematode. When we find strong positive correlations between the abundances of microbial families X and Y in a trematode species, it indicates that these two microbial families are more likely to co-occur at similar abundances among individuals of this host species. In contrast, negative correlations suggest that the two microbial families are less likely to co-occur. Non-significant correlations indicate that there are no clear co-occurrence patterns between these families. Because many of the microbial families showed zero abundance values in some individual trematodes, we used a non-parametric correlation test (i.e., Spearman’s rank correlation). We report correlation coefficients (*r*) and their statistical significances (i.e., whether *p* < 0.05 or not).

Second, we explored the relationships among correlation coefficients (*r*), which we calculated in the first step above among different trematode species. This analysis allowed us to test whether the microbial co-occurrence patterns (i.e., direction and strength) are consistent or whether they differ among the four different trematode hosts. For example, is a strong negative correlation observed between microbial families X and Y in one trematode also strong and negative in the other three trematodes? In this analysis, a significant positive relationship (close to 1:1 lines; Fig. 3) between the pairwise correlations obtained above in two trematode species would suggest that co-occurrence patterns (either positive or negative) are consistent across these two types of habitats (i.e., trematodes), whereas a significant negative relationship would suggest that co-occurrence patterns are inconsistent and highly opposite among habitats (i.e., microbial families X and Y are more likely to co-occur within trematode A, while these families are mutually exclusive within trematode B). Non-significant relationships would also suggest co-occurrence patterns are inconsistent among habitats.

We examined these relationships for all pairs of trematode species, resulting in 4(4 − 1)/2 = 6 combinations. We used Spearman’s rank correlation for this analysis. We considered *p* < 0.05 statistically significant and 0.05 < *p* < 0.1 indicative of statistical trends (i.e., marginally significant). We acknowledged that these repeated statistical tests without *p*-value corrections can lead to Type I errors. However, our focus was not on statistical significance among pairwise correlations, but rather on whether the correlative patterns were consistent across trematode identities. Therefore, we did not conduct *p*-value corrections. All statistical analysis were performed using R v. 4.3.1 [30].

## Results

**Fig. 1:**
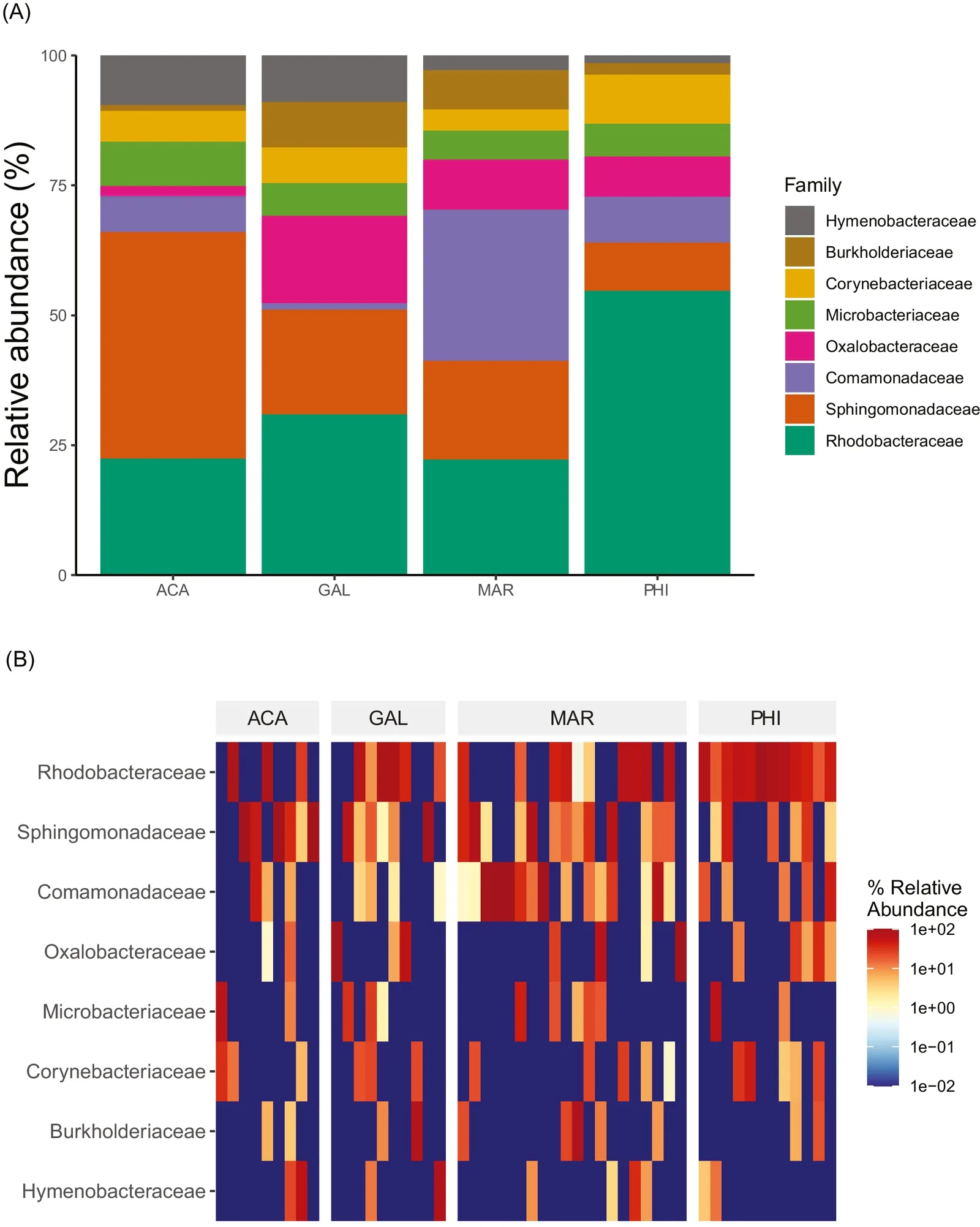
Relative abundance of the eight bacterial families associated with at least 20% of trematode individuals of each species. **A** Bar plots of bacteria families relative abundance, pooled across individuals of each trematode species based on mean relative abundance per species. **B** Heat map showing the relative abundance of the eight bacterial families (rows) in each trematode individual (columns), organized by trematode species. *ACA Acanthoparyphium*, *GAL Galactosomum*, *MAR Maritrema*, *PHI Philophthalmus*

**Fig. 2:**
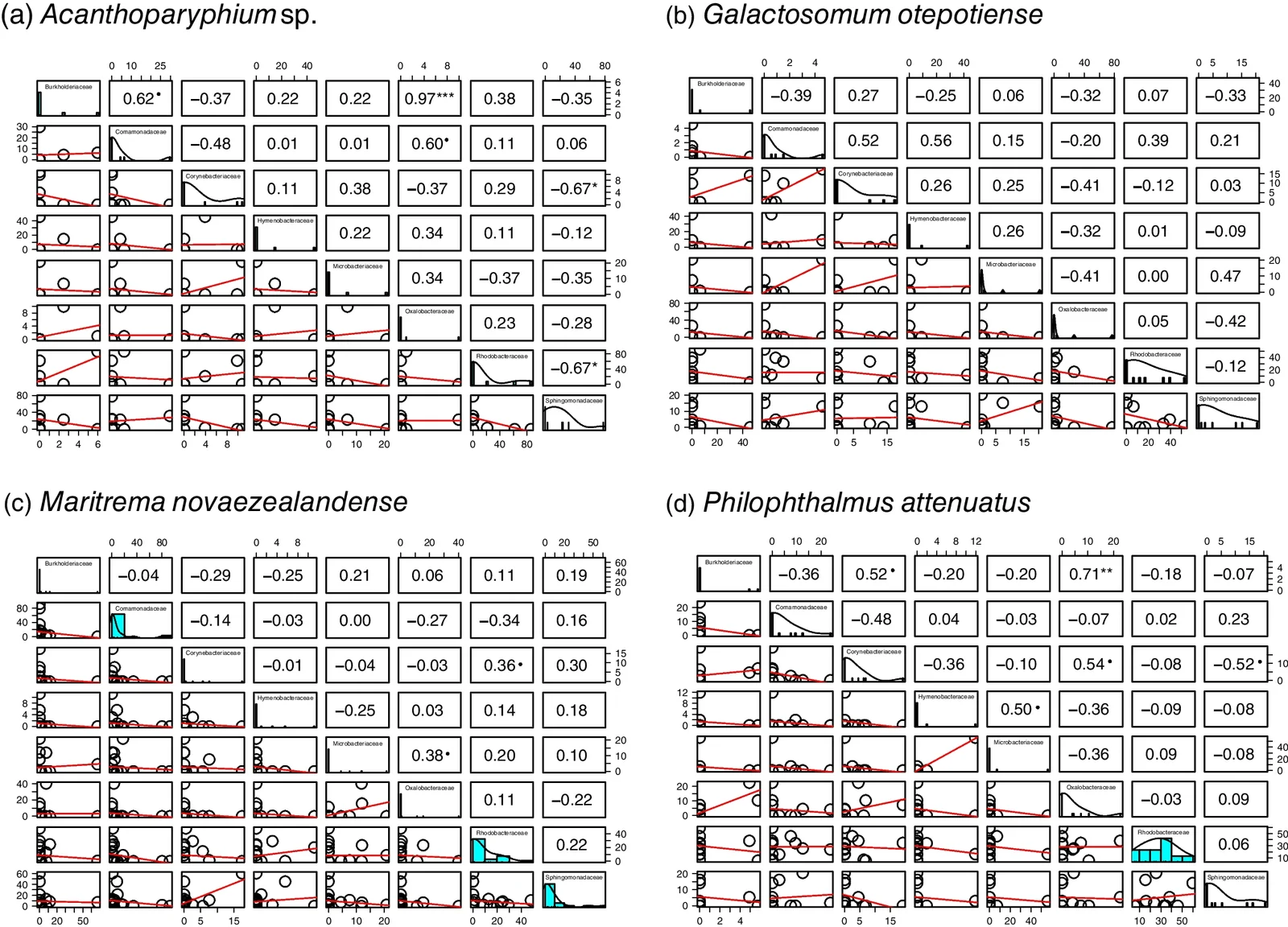
Correlations of abundance between all possible pairs of microbial families across individual trematodes in each four trematode species. **a** *Acanthoparyphium* sp., **b** *Galactosomum otepotiense*, **c**. *Maritrema novaezealandense*, **d** *Philophthalmus attenuatus*. Statistical (marginal) significances were shown as follows; ***p < 0.001, **p < 0.01, *p < 0.05, •*p* < 0.1

The compositions and relative abundance of eight bacterial families highly varied among trematode individuals and species (Fig. 1). In all four trematode species, most pairwise microbial associations, measured by the correlation between their respective abundance, were neither significant nor clear (Fig. 2). In *Acanthoparyphium* sp. and *Philophthalmus attenuatus*, we found four or more significant or marginally significant associations between microbial families (Fig. 2), whereas no significant correlations were found in *G. otepotiense* and *M. novaezealandense*, although a few marginal correlations were observed (Fig. 2)*.*

**Fig. 3:**
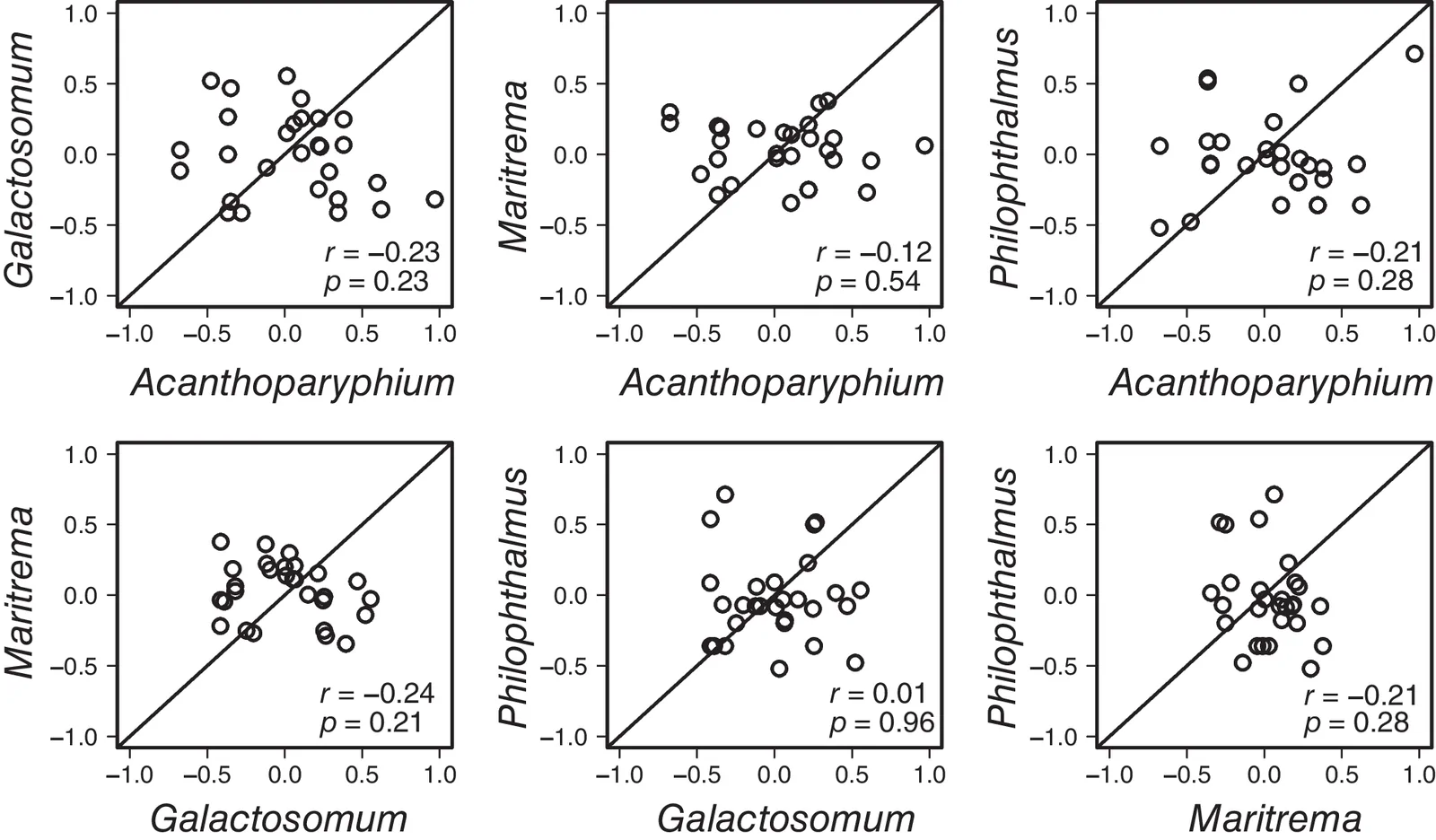
Results of the consistency tests of microbial co-occurrence patterns among four trematode hosts (six species combinations). Each point represents a Spearman’s correlation coefficient calculated for a particular pair of microbial families within each trematode species (*N* = 28 pairwise combinations among microbial families per trematode species, see Fig. 2). Lines represent the 1:1 relationship expected if co-occurrence patterns were consistent between the focal trematode species

We then tested whether the correlation coefficients from Fig. 2 correlate between trematode species to test for any consistency. Among all six pairs of trematode hosts, neither significant positive nor negative relationships were detected (Spearman’s rank correlation, all *p* > 0.10; Fig. 3) among the pairwise correlations between relative abundances of bacterial families (Fig. 3), suggesting that microbial co-occurrence patterns varied substantially and were inconsistent among host species.

## Discussion

Elucidating co-occurrence patterns and their drivers in microbial communities within animals is an essential first step toward understanding the patterns and processes of community structuring [6, 7]. Although such co-occurrence patterns can vary among habitats with different characteristics [13–15], testing whether these patterns change across habitats is generally difficult because it is challenging to achieve sufficient replication in controlled environments. Here, we examined co-occurrence patterns among different microbial families in four trematode species. As expected, even though we sampled individual trematodes that occur in the same snail species collected from a single habitat on the exact same day, we found that microbial co-occurrence patterns varied greatly among the four trematode species. This result suggests that the strength and/or direction of co-occurrence among microbial families are affected by their immediate habitat (i.e., the trematode species identity).

Different biological characteristics among trematode species can explain variations in co-occurrence patterns among microbial families. For instance, feeding modes and microhabitats have been suggested as critical determinants of microbial community structure [14, 15], and hence potential differences in these factors among trematodes may explain our results. Although all four trematodes inhabit a single snail species, each may occupy different body parts of the snail, leading to acquisition of distinct microbiomes from their hosts. Feeding modes also differ among these four species. While three trematode species (*G. otepotiense*, *P. attenuatus*, *Acanthoparyphium* sp.) inhabit the snail as rediae, possessing a mouth and actively feeding on host tissues and other co-infecting trematodes [31], *M. novaezealandense* exists as sporocyst stages, lacking a mouth and passively absorbing nutrients from the surrounding environment. However, we did not find any consistent co-occurrence patterns of microbial taxa among trematodes with the same feeding modes, nor did we find consistency in their overall microbial community compositions [18].

Another biological factor that may influence co-occurrence patterns is trematode body size, as each trematode larva shows different body sizes. For instance, the rediae of *Acanthoparyphium* sp. are 740–870 µm [32], while the sporocysts of *M. novaezealandense* are 170–250 µm [33]. In general, host (habitat) size strongly affects microbial community composition, as larger habitats (hosts) are likely to harbor a greater diversity of microbial taxa due to the relatively larger availability of resources (i.e., space and nutrients) [34, 35], which may lead to higher detection rates of co-occurrence. Indeed, we found a relatively higher number of significant and marginally significant co-occurrence patterns in *Acanthoparyphium* sp. compared to the other trematode hosts (Fig. 2), suggesting that their larger body size may have contributed to create the observed co-occurrence patterns.

Host physiological factors, particularly host immunity, may have also affected our results. Numerous studies have reported that host physiological states, including immune responses, can shape microbial community structure within hosts [8, 11]. When microbial taxa exhibit similar levels of rejection or tolerance to specific immune responses produced by their trematode hosts, these taxa are more likely to co-occur within the hosts. In contrast, when microbial taxa show differing levels of tolerance or rejection responses against host immune reactions, they are less likely to co-occur in the same hosts. Although no direct information on immune responses within trematodes is available, several previous studies on trematode hosts have discussed the potential effects of host-specific physiology (immunity) on the microbiome [18]. Given that the four trematode species examined are phylogenetically distinct (i.e., each species belongs to a different family), their immune systems likely differ, which may have influenced our observed patterns.

Although trematodes primarily acquire microbiomes from the same regional pool (i.e., the microbial communities within the snail and their surrounding habitats) because they shared the same immediate environments (i.e., the same snails and habitats), each trematode host can acquire microbial families at different times and/or in a different order (e.g., [36, 37]), mainly due to vertical transmission. These differences in assembly timing play an important role in shaping microbial communities (e.g., [36, 37]) and could therefore influence co-occurrence patterns. While all four trematodes were collected from the same snail species, their other developmental stages (i.e., metacercariae, adults) infect different host species or individuals [19], where trematodes can actively or passively acquire distinct microbiomes. As demonstrated by Jorge et al. [29, 38], microbiomes in trematodes can be vertically transmitted across multiple developmental stages, suggesting that similar vertical transmission likely occurs in our study system. Additionally, infection periods for each trematode species can vary among individuals since snails acquire trematode infections at different times. Such differences in infection timing among trematode individuals could also affect our results.

Co-occurrence patterns can provide the first line of evidence of biotic associations among microbial taxa, such as facilitation or competition ([39], but see [40]). Although such biological associations may not be common in our study systems, as most of the observed correlations were not significant (at least at family taxonomic level), co-occurrence patterns may still be partly driven by these biotic associations. Indeed, many studies have identified bacterial associations as relatively common within host organisms [14, 37, 40] and as potentially major drivers of community composition. However, correlations in microbial relative abundances do not necessarily imply biotic interactions among these groups [39, 40], and careful consideration is required when discussing possible biological associations among bacterial taxa. Future studies, such as experimental or longitudinal studies, which are useful for identifying causal relationships, are necessary to determine whether the observed co-occurrence patterns reflect biological associations.

We acknowledge several limitations in our study. Although we tried to minimize the effects of immediate environments by sampling the same snail species from a single location on the same day, potential differences in body size, age, nutritional states, and microhabitats among individual snails could not be controlled. These subtle differences among snails (habitats) could affect our results, and further studies are required to determine whether host identity is the primary factor in shaping the observed co-occurrence patterns. Additionally, many microbial families showed zero relative abundance, even though we focused on microbial family groups with relatively high abundance that were prevalent among all four trematode species (see the “Methods” section). This limitation made some pairwise comparison among microbial families unsuitable for testing co-occurrence. Furthermore, our focus on the family-level patterns of bacterial associations also has potential problems. That is, statistical significance of family-level patterns of bacterial associations compared among the different trematode species is not the same as biological significance. The latter assumes that bacterial taxa within the same family share some functional properties, an assumption that would require testing. Nonetheless, bacterial lineages belonging to the same family likely have similar metabolic routes, and potentially similar biological functions [28], and thus, using taxonomic levels at the family and above remains an informative approach in microbiome studies (e.g., [17, 37, 41]). Despite these potential limitations, our study clearly showed that co-occurrence patterns of microbial families varied among trematode individuals and species, using a highly controlled study design compared to previous studies. Thus, we believe that our conclusions remain valid.

In summary, we showed highly variable microbial co-occurrence patterns among four different trematode hosts, even after controlling immediate environmental factors as much as possible. Our results suggest that the abundance of particular microbial taxa is generally independent of the abundance of other taxa, and that co-occurrence between pairs of taxa is not strong enough to persist across contexts. Identifying key determinants and processes governing these inconsistent co-occurrence patterns depending on habitats (the host species) is a required next step to understand how biological communities assemble in nature. This is a particularly important step for microbial communities because microbes have profound effects on host animals, including humans (e.g., [42]). Recently, several key microbial taxa, which have either positive or negative effects on their hosts, have been identified (e.g., [43]). Revealing key determinants of microbial associations would help us to control these key microbial taxa, and thus contribute to many study areas, such as resource conservation and human health.

## Data Availability

Raw sequence reads are available in the SRA (BioProject PRJNA972185, BioSamples SAMN35067136 to SAMN35067307); bioinformatics scripts, filtered data, FastQC reports and metadata are available on Figshare (https://doi.org/10.6084/m9.figsh are.22881482).

## Code Availability

The R code for statistical analyses and showing figures used in the current study are available from the corresponding author on reasonable request.
